# Forest Succession Shapes Soil microbial Communities through Region-specific Edaphic Filters in Tropical and Subtropical Forests

**DOI:** 10.1007/s00248-026-02734-1

**Published:** 2026-03-23

**Authors:** Waseem Muhammad, Xinyu Zhou, Xiaocheng Yu, Kechen Yang, Rong Bai, Leiyun Feng, Qiang Luo, Zhiqing Zhou, Cong Wang, Jingchao Li, Kui Ji, Hua-Zheng Lu

**Affiliations:** 1 Yunnan Key Laboratory of Forest Ecosystem Stability and Global Change, Xishuangbanna Tropical Botanical Garden, Chinese Academy of Sciences, Mengla, China; 2 Xishuangbanna Station for Tropical Forest Ecosystem Studies, Chinese Academy of Sciences, Mengla, China; 3 School of Life Science, University of Chinese Academy of Sciences, Beijing, China; 4 College of Landscape Architecture and Forestry, Shandong Key Laboratory for Germplasm Innovation of Saline-Alkaline Tolerant Grasses and Trees, Qingdao Agricultural University, Qingdao, P.R. China; 5 Schools of Soil and Water Conservation, Southwest Forestry University, Kunming, China; 6 Institutes of Microbiology, Chinese Academy of Sciences, Beijing, China; 7 Xishuangbanna National Nature Reserve, Mengla, 666300 China

**Keywords:** Forest succession, Microbial diversity, Bacterial and Fungal Community composition, Soil properties, Soil depth, Pakistan & China

## Abstract

Understanding how soil microbial communities respond to forest succession is essential for predicting ecosystem functions and biogeochemical stability. We investigated bacterial and fungal communities across three successional stages (early, mid, late) and three soil depths (0–10, 10–20, 20–50 cm) in forests of Pakistan and China using high-throughput amplicon sequencing of 108 soil samples. This cross-regional, depth-resolved study aimed to determine whether microbial successional trajectories and soil–microbe relationships are general or region-specific. Preliminary results showed that the forest succession was accompanied higher soil organic carbon (SOC), total nitrogen (TN) and declined soil pH in Pakistan. It indicates consistent acidification and potential phosphorus limitation in mature stands. Whereas SOC and total potassium (TK) exhibited mid-successional peaks in China, that indicates different resource/nutrient dynamics. Mid-forest successional stages showed maximum bacterial diversity, whereas late succession revealed the highest fungal diversity (kingdom-specific responses). Community composition shifted from copiotrophic taxa in early stages to oligotrophic taxa in mature forests. Soil pH was the most influential factor shaping microbial composition in Pakistan, whereas potassium availability was the most influential factor in China. These cross-regional, depth-resolved results reveal both successional patterns and region-specific environmental controls, offering new insights into microbial community composition during forest development and providing guidance for forest restoration and soil-carbon management across biogeographically diverse regions.

**Supplementary Information:**

The online version contains supplementary material available at 10.1007/s00248-026-02734-1.

## Introduction

Forest succession is closely related to the dynamics of terrestrial ecosystem structure and function [1]. Forest ecosystems are dynamic and constantly changing due to succession, which alters the vegetation and strongly affects the soil and microbial communities below ground. Microbial communities, such as bacteria, fungi, and other microorganisms are involved in key ecosystem processes, including decomposition of organic matter, nutrient cycling, and soil structure formation [2]. These microbial communities are not constant, they change over time in response to shifts in plant composition, soil properties, and soil depth. Understanding the dynamics of microbial diversity and community structure during forest succession is essential for predicting ecosystem responses to environmental change and for implementing more effective forest-management strategies [3].

Forest succession generally has a positive impact on soil microbial diversity and community composition. However, the effects can vary depending on the succession stage and specific microbial patterns of the soil [4]. Copiotrophic r-strategists predominate in the early stages of succession and encompass taxa that can capitalize on favorable organic conditions. However, when organic matter becomes more complex and accumulates copiotrophic r-strategists shift towards oligotrophic K-strategists, including fungi and bacteria specialized for degrading lignin and other recalcitrant molecules [5]. Such changes in microbial community structure are usually caused by soil properties, such as pH, organic carbon (C), nitrogen (N), phosphorus (P), and potassium availability, as well as the quality of organic matter, and variation in environmental conditions in the soil. Soil pH is especially important in structuring microbial communities: some bacteria, including *Acidobacteriota*, prefer acidic soils, whereas others grow better in neutral or alkaline environments [6]. Carbon and nitrogen are key nutrients that support microbial growth; their availability determines the dominance of various groups of microbes [7]. Potassium, though less frequently limited, is useful for controlling microbial activity in low nutrient soils [8]. Phosphorus is essential for microbial communities and its concentration reduces with time due to the fixation in minerals in mature soils [9]. The availability of phosphorus in the soil can be further decreased by soil acidification in tropical and subtropical regions in such a way that availability of phosphorus becomes less available to microbes. The carbon to phosphorus (C: P) and nitrogen to phosphorous (N: P) ratios are critical for understanding microbial nutrient limitations. These ratios change as the forest matures, where phosphorus is also a limiting factor in older forests, which affects the overall structure of the microbial communities [10].

Several studies have documented changes in microbial diversity and community composition during forest succession, often reporting increased diversity and a shift from fast-growing copiotrophic taxa in early stages to slower-growing oligotrophic taxa in mature forests [4, 11]. However, the strength and direction of these soil–microbe relationships may differ between regions with contrasting climates and soil types. Moreover, most research has focused on single regions, limiting our ability to determine whether successional microbial trajectories are generalizable or region-specific. In particular, the strength of soil–microbe relationships has been shown to change during succession, but it is unclear whether these relationships weaken during transitional mid-successional stages when soil resources and plant communities are rapidly changing. Several studies suggest that mid-successional stages represent a transitional phase characterized by rapid changes in vegetation inputs, nutrient availability, and microbial functional traits, potentially weakening consistent soil–microbe linkages compared to early or late stages [12, 13]. These inconsistent findings highlight the need for further research to understand how these factors interact in various stages of forest succession and in different climatic regions.

Therefore, this study evaluates the successional dynamics in two climatically different regions i.e., subtropical forests in Pakistan and tropical forests in China to test whether successional microbial trajectories/patterns and soil–microbe relationships are general or region-specific. These regions were selected because they represent contrasting environmental filters (arid vs. humid climate, alkaline vs. acidic soil) that allow us to evaluate the robustness of successional patterns under different constraints. Chinese tropical forests receive high rainfall, warm climate and have acidic soils, whereas the subtropical forests in Pakistan experience seasonal variation and have alkaline soil pH. Analyses were performed within each region to examine how microbial communities and soil–microbe relationships reshape along successional stages, highlighting patterns that are either consistent across regions or specific to local conditions. It was hypothesized that (1) microbial community composition shifts across forest successional stages with copiotrophic r-strategists taxa dominating early stages and acid-tolerant oligotrophs k-strategists taxa increasing as forests mature and nutrients change, and (2) the strength of the associations between microbial diversity, microbial communities and soil properties varies across forest successional stages with weaker relations expected during the mid-successional stage when communities transition in functional traits. To test these research hypotheses, bacterial and fungal diversity along with community composition, and their relationships with soil properties were analyzed across different (early, mid and late) successional stages using high-throughput sequencing (16 S rRNA and ITS) combined with detailed biogeochemical analyses.

## Materials and Methods

### Study Sites and Soil Sampling

This study was conducted across six forest sites representing early, mid, and late-successional stages across Pakistan and China. Successional stages were classified based on stand development following disturbance, as described by Oliver [14].

Three successional stages (Garhi Chandan Forests, Malakand Division Forests, and Ayubia National Park**)** were selected from Pakistan. Garhi Chandan Forests (G_C), one of the earliest succession artificial stages formed in 2014 as part of the Billion Tree Tsunami afforestation project in Pakistan. The forest is situated close to Peshawar (33°50′N, 71°42′E; elevation 494 m, 3,141.6 hectares) and is arid with summer temperatures reaching 45 °C and winter temperatures reaching 4 °C. Some of the common drought-tolerant plant species that are dominant in this forest are *Acacia modesta*, *Ziziphus mauritiana*,* Tamarix spp.*,* Eucalyptus spp.*,* Populus spp*., and *Moringa oleifera*. The Malakand Division Forests (M_F) is a secondary mid-successional stage situated in a subtropical dry temperate region (34°29′N, 71°55′E) with elevations ranging from 500 to 7708 m. The climate is classified as warm and temperate (Csa), with an average annual temperature of 19.9 °C and rainfall of 743 mm. The soil is moist and loamy, and the region supports extensive coniferous and broadleaf forests. Ayubia National Park (A_N) represents a late-successional temperate primary forest located in the Abbottabad District (34°01′N, 73°24′E). Designated in 1984, this protected area spans 312 ha at elevations from 1220 to 2865 m. The region hosts subalpine meadows and moist temperate forests and receives over 1500 mm of precipitation annually, including monsoonal rain and winter snowfall.

Three successional stages in Menglun Township Xishuangbanna were selected from China. Rubber Plantation (21°55.51′N, 101°16.02′E), an early-successional anthropogenic stage dominated by *Hevea brasiliensis*. The site lies between 517 and 1541 m elevation and experiences a tropical monsoon climate with average temperatures of 20–22.5 °C and annual rainfall of 1200 to 1800 mm [15]. Secondary Forest (S_F) (21°55.39′N, 101°16.07′E), a mid-succession stage characterized by semi-deciduous tropical vegetation. The region is characterized by acidic, brick-red soils and distinct dry and wet seasons shaped by the southwest monsoon season. Primary Forest (P_F) (21°55.06′N, 101°11.09′E) is a late-successional stage tropical rainforest representing the climax community in Xishuangbanna. This site harbors high structural complexity and biodiversity and serves as a reference for undisturbed tropical forest ecosystems.

For soil sampling, three independent stands per successional stage were selected to ensure true replication in both regions. Within each stand we established two 20 × 20 m plots placed randomly, and five sub-core points per plot were sampled at ~ 5 m spacing. Soils were collected from three depth layers (0–10 cm, 10–20 cm, 20–50 cm), the five sub-cores from each plot × depth were homogenized into a single composite sample to represent the plot-level soil. Using this five-point sampling method, a total of 18 composite soil samples were obtained from each successional stage, yielding 108 soil samples overall (2 regions × 3 successional stages × 3 stands × 2 plots × 3 layers). The samples were sieved through a 2.00 mm mesh to remove stones, roots, and macrofauna. Samples were immediately sealed in labeled plastic bags. Pakistani soil samples were transported within one week in ice-packed insulated boxes (0–4 °C) to Xishuangbanna Tropical Botanical Garden (XTBG), Chinese Academy of Sciences. Upon arrival, samples were divided into two portions: one for physicochemical analyses, stored at 4 °C, and another for high-throughput DNA sequencing, immediately preserved at − 80 °C. DNA integrity was verified prior to sequencing to ensure that microbial community structure was maintained during transport.

### Soil Properties Analysis

Air-dried and sieved (2 mm) soil samples were analyzed for their key physicochemical properties. Soil pH was measured using a calibrated pH meter with a glass electrode in a 1:2.5 soil-to-water suspension. Briefly, 10 g of air-dried soil was mixed with 25 mL of deionized water, stirred, and allowed to equilibrate for 30 min prior to measurement. All measurements were performed duplicate or triplicate with appropriate blanks, calibration standards, and quality control samples to ensure analytical accuracy and reproducibility. Soil organic carbon (SOC) was determined following the dichromate oxidation method (LY/T 1237–1999), in which 0.5 g of soil was oxidized using 0.4 mol L⁻¹ potassium dichromate (K₂Cr₂O₇) and concentrated sulfuric acid (H₂SO₄), followed by heating at 135–140 °C for 5 min. The remaining dichromate was titrated with 0.2 mol L⁻¹ ferrous sulfate (FeSO₄) using diphenylamine as an indicator of the reaction completion. The organic matter content was estimated to multiply the SOC by a factor of 1.724. Total nitrogen (TN) was quantified using a Vario Isotope Cube elemental analyzer (Elementar Analysensysteme GmbH, Germany) following the Dumas combustion method in accordance with LY/T 1228–2015. Approximately 20–50 mg of finely ground soil was combusted in an oxygen-rich atmosphere, and nitrogen content was measured from the resulting gases.

Total phosphorus (TP) and total potassium (TK) were determined after acid digestion with a mixed solution of nitric acid (HNO₃), perchloric acid (HClO₄), and hydrofluoric acid (HF), following the standards LY/T 1232–2015 and LY/T 1234–2015, respectively. For each analysis, 0.2 g of soil was digested, and the resulting solution was analyzed using inductively coupled plasma atomic emission spectrometry (ICP-AES; iCAP 7400, Thermo Fisher Scientific, USA). The same digestion procedure was used for both TP and TK, with element-specific emission wavelengths applied during ICP-AES measurements.

### DNA Extraction, PCR Amplification, and Illumina Sequencing

Soil genomic DNA was extracted using the MagBeads FastDNA Kit for Soil (MP Biomedicals, Cat. No. 116564384, CA, USA) following the manufacturer’s protocol. DNA concentration and purity were measured with a NanoDrop NC2000 spectrophotometer (Thermo Fisher Scientific, Waltham, MA, USA), and integrity was verified by agarose gel electrophoresis. For bacteria, the 16 S rRNA gene V3–V4 region was amplified using primers 338 F (5′-ACTCCTACGGGAGGCAGCA-3′) and 806R (5′-GGACTACHVGGGTWTCTAAT-3′), yielding an expected product of ~ 460 bp. For fungi, the ITS1 region was amplified using primers ITS1F (5′-CTTGGTCATTTAGAGGAAGTAA-3′) and ITS2 (5′-GCTGCGTTCTTCATCGATGC-3′). PCR amplification was performed using a high-fidelity DNA polymerase (Q5^®^ High-Fidelity DNA Polymerase, New England Biolabs, Ipswich, MA, USA) in a 25 µL reaction system with sample-specific dual barcodes. Thermal cycling for the 16 S rRNA gene consisted of: initial denaturation at 98 °C for 30 s; 25 cycles of 98 °C for 10 s, 55 °C for 20–30 s, and 72 °C for 20–30 s; followed by a final extension at 72 °C for 2–5 min. Fungal ITS amplification used 28–30 cycles with annealing at ~ 52–55 °C. PCR products were purified with VAHTS™ DNA Clean Beads (Vazyme, Nanjing, China), quantified with the Quant-iT PicoGreen dsDNA Assay Kit (Invitrogen, Carlsbad, CA, USA), and pooled in equimolar amounts. Sequencing was performed on the Illumina MiSeq platform (2 × 250 bp, MiSeq Reagent Kit v3) at Shanghai Personal Biotechnology Co., Ltd. (Shanghai, China). Negative extraction and PCR controls were included throughout library preparation and sequencing; controls yielded negligible read counts (≪1% of sample reads) and did not affect downstream analyses.

### Sequence Data Processing

Bioinformatic processing of bacterial 16 S rRNA and fungal ITS reads were performed in QIIME2 v4.3.3 [16], using the DADA2 workflow to infer amplicon-sequence variants (ASVs). Raw paired-end FASTQ files were demultiplexed with the QIIME2 demux plugin, and primers were trimmed with cutadapt v5.1 [17]. Reads were quality-filtered, denoised, merged, and chimeras were identified and removed during the DADA2 step [18]. Non-singleton ASVs were aligned with MAFFT v7 [19], and a midpoint-rooted phylogenetic tree was built with FastTree2 v2.1.11 [20]. Taxonomy was assigned to ASVs using the classify-sklearn naïve-Bayes classifier in the QIIME2 feature-classifier plugin [21], trained on the SILVA v138 reference database for bacterial 16 S rRNA genes and the UNITE v9.0 database for fungal ITS sequences.Alpha-diversity indices, including Chao1 richness [22], Shannon diversity [23], and others as appropriate, were calculated with the QIIME2 diversity plugin. Beta-diversity was assessed with Bray–Curtis and Jaccard distance metrics and visualised using non-metric multidimensional scaling (NMDS).

### Statistical Analysis

All statistical analyses were conducted in R (v4.3.3). To analyze soil properties across successional stages and depths, a linear mixed model was used to evaluate the main effects of country, successional stage and soil depth, as well as their interactions. Post-hoc comparisons were carried out using Tukey’s HSD test to identify significant differences between depth categories. Statistical significance for the successional stage factor was assessed based on p-values, with significance indicated by stars, while differences between depth levels were shown with letter groupings.

Linear mixed models (LMMs) with Tukey’s HSD were employed to evaluate the effects of Country, Successional Stage, and Soil Depth on bacterial and fungal alpha diversity. Successional Stage, Country, and Depth were treated as fixed effects, and Plot nested within Stand was included as a random effect (Stand/Plot). ANOVA (Type III) was then performed on the model to evaluate the significance of the fixed factors and their interactions. The analysis was conducted using the “lmerTest” package in R, with assumptions of normality and homogeneity of variance accessed via the “performance” package. In addition, Boxplots were used to visualize microbial diversity across successional stages and soil depths. Wilcoxon rank-sum tests were employed for comparisons between successional stages and depth categories within each successional stage, with p-values denoted by stars for stages and letter groupings for Depth to indicate statistical differences. Custom color palettes were applied to distinguish depth categories, and stages were faceted by diversity index and microbial type (Bacteria vs. Fungi).

We calculated relative abundances of bacterial and fungal taxa and visualized them with stacked bar plots. Data were normalized before comparing distributions across successional stages and depths. Non-Metric Multidimensional Scaling (NMDS) was performed on beta diversity data to analyze community composition across successional stages. Abundance data were transformed using Hellinger’s method for standardization, followed by Bray-Curtis dissimilarity to generate distance matrices. Ordinations were visualized with ggplot2 for clarity. To assess statistical significance of clustering patterns across ecological layers and environmental gradients, PERMANOVA (adonis2) was conducted, providing insights into the spatial distribution and diversity of microbial and fungal communities.

Mantel tests were used to assess the correlation between soil properties and microbial diversity metrics (both alpha and beta diversity) for bacteria and fungi. Spearman’s rank correlation was applied to examine relationships between soil variables, and correlation matrices were visualized using the qgraph package to highlight significant associations. Statistical significance was evaluated using permutation-based testing with p-values reported accordingly. Redundancy Analysis (RDA) was employed to explore the relationship between environmental variables and microbial communities, with results visualized through biplots that included successional stages and soil depth as factors. Environmental data were standardized using the scale function, and the variance explained by each axis in the RDA was calculated. Stages were clustered using ellipses, colored by successional stages and shaped by soil depth, with significant environmental variables indicated by arrows on the plots.

## Results

### Soil Physicochemical Properties across Successional Stages and Depths

Soil properties differ significantly across succession in both regions (Table S1). Soil organic carbon (SOC), Total nitrogen (TN), carbon to nitrogen ratio (C: N), carbon to phosphorus ratio (C: P) and nitrogen to phosphorus ratio (N: P) increased with forest succession stages in Pakistan. Total phosphorus (TP) and Total potassium (TK) showed a different trend, TP was significantly higher in mid-successional stage (M_F) as it first increased from early to mid-stage and then decreased from mid to late stage, while TK first decreased from early to mid-stage and then increased from mid to late stage in Pakistan. Soil pH decreased across successional stages showing a decreased from G_C (8.21 ± 0.30) to A_N (5.66 ± 0.21). Across succession stages, soil depth had no significant impact on the physicochemical properties In Pakistan.

Across successional stages, soil organic carbon (SOC), Total potassium (TK), carbon to nitrogen ratio (C: N), carbon to phosphorus ratio (C: P) and soil pH first increased from early to mid-stage and then decreased from mid to late successional stage In China. Total nitrogen (TN) and Total phosphorus (TP) increased with forest succession stages, while nitrogen to phosphorus (N: P) continuously decreased with forest succession stages In China. Across forest succession stages, soil depth show significant effects, especially in R_P and S_F forest succession stages, where the concentration of various soil nutrients changed with depth in China.

### Microbial Alpha Diversity across Successional Stages and Soil Depths

**Fig. 1:**
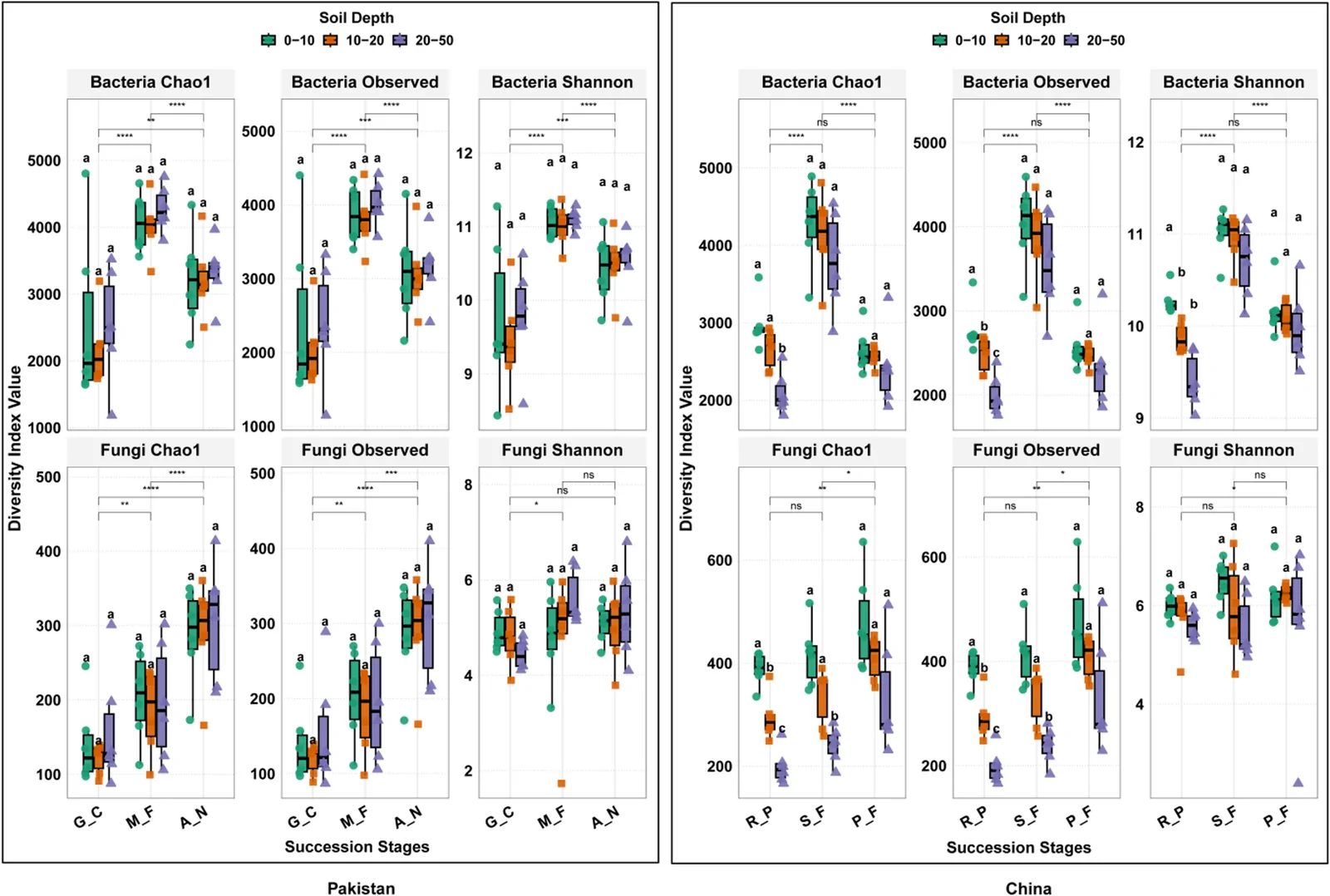
Alpha diversity of soil bacterial and fungal communities across successional stages and soil depths in Pakistan and China (means ± SE, *n* = 6). Boxplots illustrate the variation in bacterial and fungal diversity indices (Chao1, Observed OTUs, and Shannon) across different forest succession stages (G_C, M_F, A_N in Pakistan; R_P, S_F, P_F in China) and soil depths (0–10 cm, 10–20 cm, and 20–50 cm). Statistical *p* < 0.01 (*), *p* < 0.001 (**), *p* < 0.0001 (***), and not significant (ns). Letters denote post hoc groupings where applicable

Soil microbial alpha diversity varied across successional stages in both countries (Fig. 1; Table 1). Across forest succession stages, bacterial alpha diversity, as measured by Chao1, observed species, and Shannon indices, was highest in the mid-successional stage, followed by the late-successional stages, and lowest in the early-successional stages in both countries. Fungal diversity, on the other hand, was highest in late-successional, showing increasing trend across successional stages for fungi.

**Table 1:** Influence of successional stages and soil depth on microbial diversity in Pakistani and Chinese forest succession stages (*n* = 6)

Country		Bacteria									Fungi									
	Effect	Bacteria Shannon			Bacteria Chao1			Bacteria Observed species			Fungi Shannon			Fungi Chao1			Fungi Observed species			
		χ^2^	DF	*P*	χ^2^	DP	*P*	χ^2^	DP	*P*	χ^2^	DP	*P*	χ^2^	DP	*P*	χ^2^	DP	*P*	
**Pakistan**	**Stages**	16.7	2	< 0.001	15.1	2	< 0.001	16.3	2	< 0.001	0.3	2	> 0.05	15.1	2	< 0.001	15.4	2	< 0.001	
	**Depth**	3.2	2	> 0.05	3.6	2	> 0.05	3.7	2	> 0.05	1.6	2	> 0.05	2.9	2	> 0.05	2.4	2	> 0.05	
	**Stages x Depth**	1.8	4	> 0.05	2.1	4	> 0.05	1.7	4	> 0.05	6.3	4	> 0.05	1.8	4	> 0.05	1.6	4	> 0.05	
**China**	**Stages**	33.1	2	< 0.001	51.2	2	< 0.001	50.6	2	< 0.001	1.3	2	> 0.05	6.6	2	< 0.05	6.7	2	< 0.05	
	**Depth**	69.5	2	< 0.001	44.6	2	< 0.001	36.4	2	< 0.001	0.9	2	> 0.05	36.8	2	< 0.001	36.9	2	< 0.001	
	**Stages x Depth**	23.7	4	< 0.001	13.1	4	< 0.001	9.2	4	> 0.05	2.1	4	> 0.05	1.1	4	> 0.05	1.1	4	> 0.05	

However, we observed no significant differences in either bacterial or fungal diversity across the three soil depths (0–10 cm, 10–20 cm, 20–50 cm) in Pakistani successional stages, as indicated by non-significant comparisons (ns) across all depths within each successional stage. In Chinese forest succession stages, both forest successional stage and soil depth influenced microbial community structure, whereas, microbial diversity is more strongly influenced by successional stages than by soil depth in Pakistan. These differences in microbial diversity patterns are consistent with differences in forest development histories, climatic conditions, and belowground ecological dynamics between the two regions.

### Microbial Community Composition across Successional Stages and Soil Depths

**Fig. 2:**
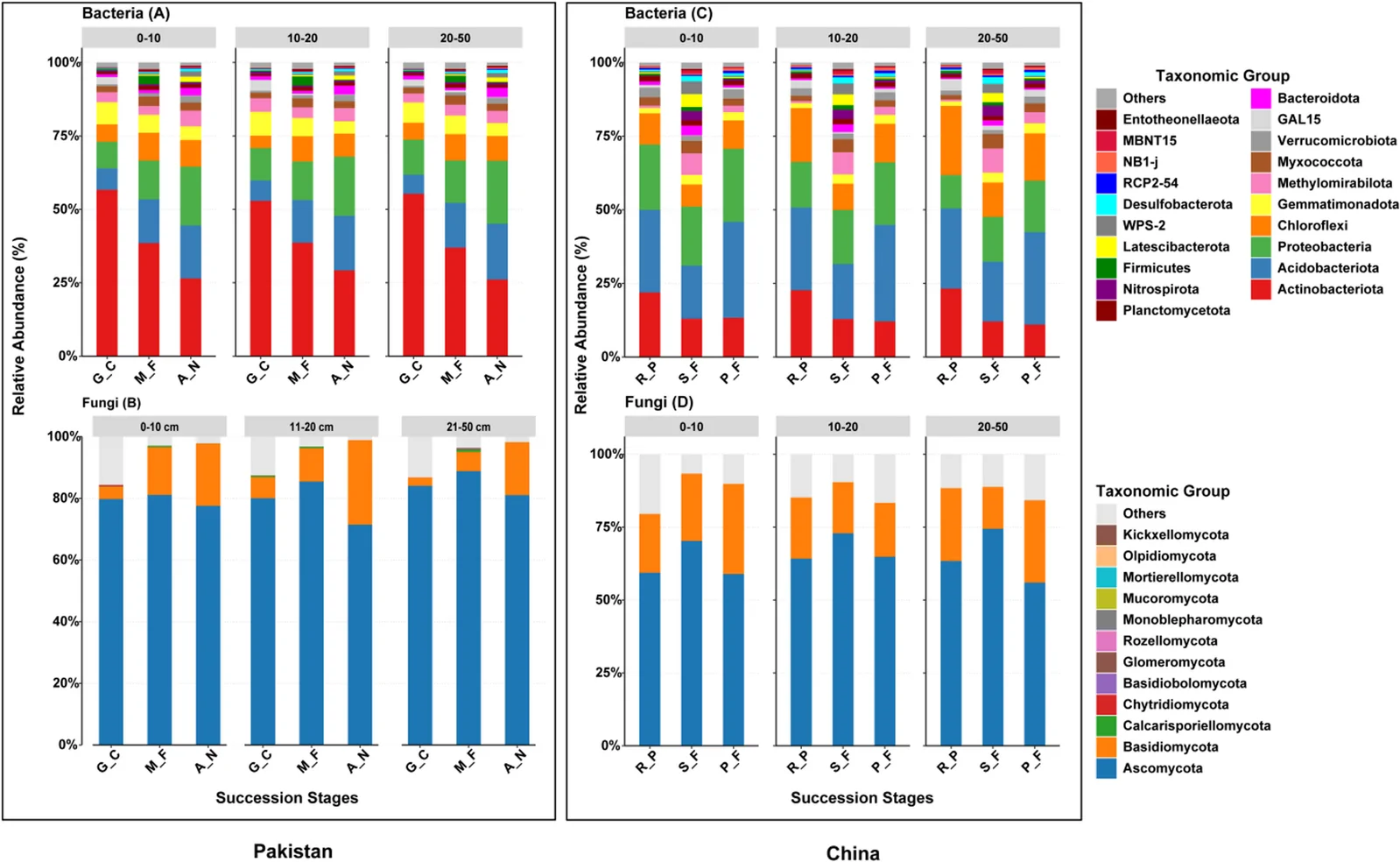
Taxonomic composition of soil bacterial and fungal communities across successional stages and soil depths in Pakistan and China (mean relative abundance, *n* = 6). Stacked bar plots show the relative abundance of major bacterial (A, C) and fungal (B, D) phyla in different successional stages (G_C, M_F, A_N in Pakistan; R_P, S_F, P_F in China) and soil depths (0–10 cm, 10–20 cm, 20–50 cm). The color legend indicates key taxonomic groups

Taxonomic profiling of microbial communities across forest successional stages revealed differences in community composition in Pakistan and China. *Actinobacteriota*,* Acidobacteriota*,* Proteobacteria* and *Chloroflexi* were the most dominant bacterial phylum in Pakistan (Fig. 2A). *Actinobacteriota* with the highest relative abundance found in early-successional stage (G_C) at the 0–10 cm depth, gradually decreasing in mid (M_F) and late-successional stage (A_N). In contrast, *Acidobacteriota*,* Proteobacteria* and *Chloroflexi* exhibited a different pattern, with their relative abundance increasing from early to mid-succession and peaking in late-successional stages. Bacterial community showed greater taxonomic diversity in Chinese succession stages (Fig. 2C). *Actinobacteriota*,* Acidobacteriota*,* Proteobacteria* and *Chloroflexi* were consistently dominant across all successional stages and depths. *Actinobacteriota* showed a continuous decline across successional stages, while *Proteobacteria* continuously increased across successional stages. The relative abundance of *Acidobacteriota* and *Chloroflexi* decreased from early to mid-succession (R_P to S_F), then increased again in late-successional stage (P_F) in China.

Fungal communities (Fig. 2B and D) were dominated by *Ascomycota* in both countries, though its relative abundance was lower in Chinese successional stages than in Pakistani forest successional stages. In both countries successional stages, *Ascomycota* had its highest relative abundance in mid-successional stage, with a gradual decrease in late-successional stages. *Basidiomycota* showed a slight increase in relative abundance with soil depth, particularly in the 20–50 cm layer. The fungal community composition in Chinese successional stages showed variation with soil depth compared to Pakistan, where *Ascomycota* dominated across all depths and successional stages.

**Fig. 3:**
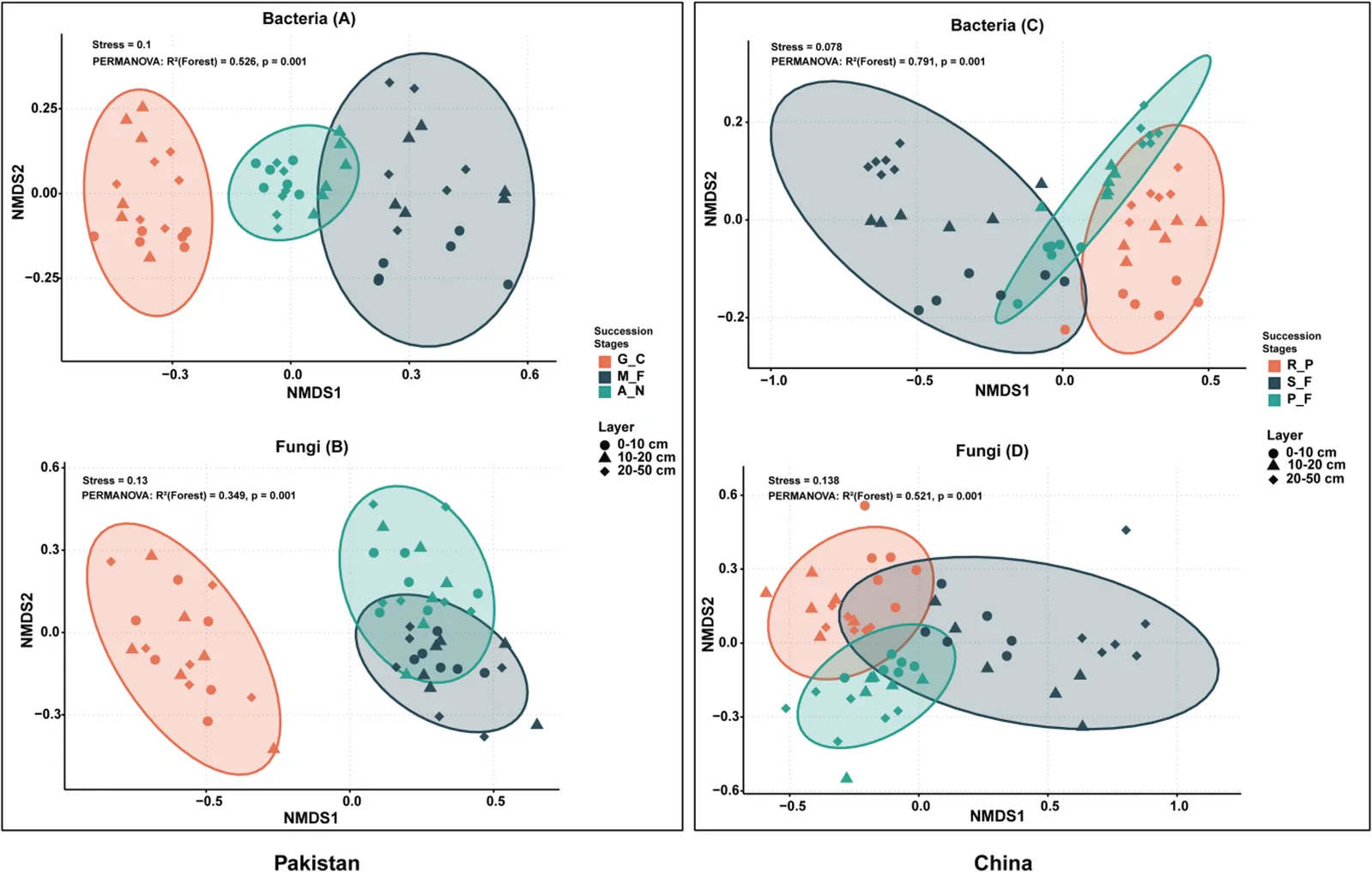
Non-metric multidimensional scaling (NMDS) ordination plots showing differences in soil microbial community composition across successional stages and soil depths in Pakistan (Panels **A–B**) and China (Panels **C–D**) (*n* = 18). Ordinations are based on Bray–Curtis dissimilarities of bacterial (A, C) and fungal (B, D) ASVs-level community data. Colored ellipses represent 95% confidence intervals for successional stages: G_C (early), M_F (mid), and A_N (late) for Pakistan; R_P (early), S_F (mid), and P_F (late) for China. Soil depth layers (0–10 cm, 10–20 cm, 20–50 cm) are indicated by point shapes (circle, triangle, diamond). PERMANOVA results show the proportion of community variation explained by Successional Stages (R²) and associated significance (*p* = 0.001 in all cases). Stress values indicate ordination reliability

NMDS ordinations based on Bray-Curtis dissimilarities showed clear successional differentiation of soil microbial communities (Fig. 3 & Table S2) in both countries. Bacterial communities clustered clearly by successional stage (PERMANOVA R² = 0.584–0.791, *R* = 0.567–0.532, *P* = 0.001; Stress = 0.078–0.10), with early-successional stage in Pakistan and mid-successional stage in China forming the most distinct groups. Fungal communities were also significantly structured by succession (R² = 0.349–0.520, *R* = 0.519–0.453, *P* = 0.001; Stress = 0.13–0.14), with transitional assemblages emerging at the same stages. These results show that forest succession is the primary ecological filter shaping microbial community composition, exerting stronger influence than soil depth.

### Soil Properties Shaping Microbial Diversity and Composition: Mantel Correlation Insights

**Fig. 4:**
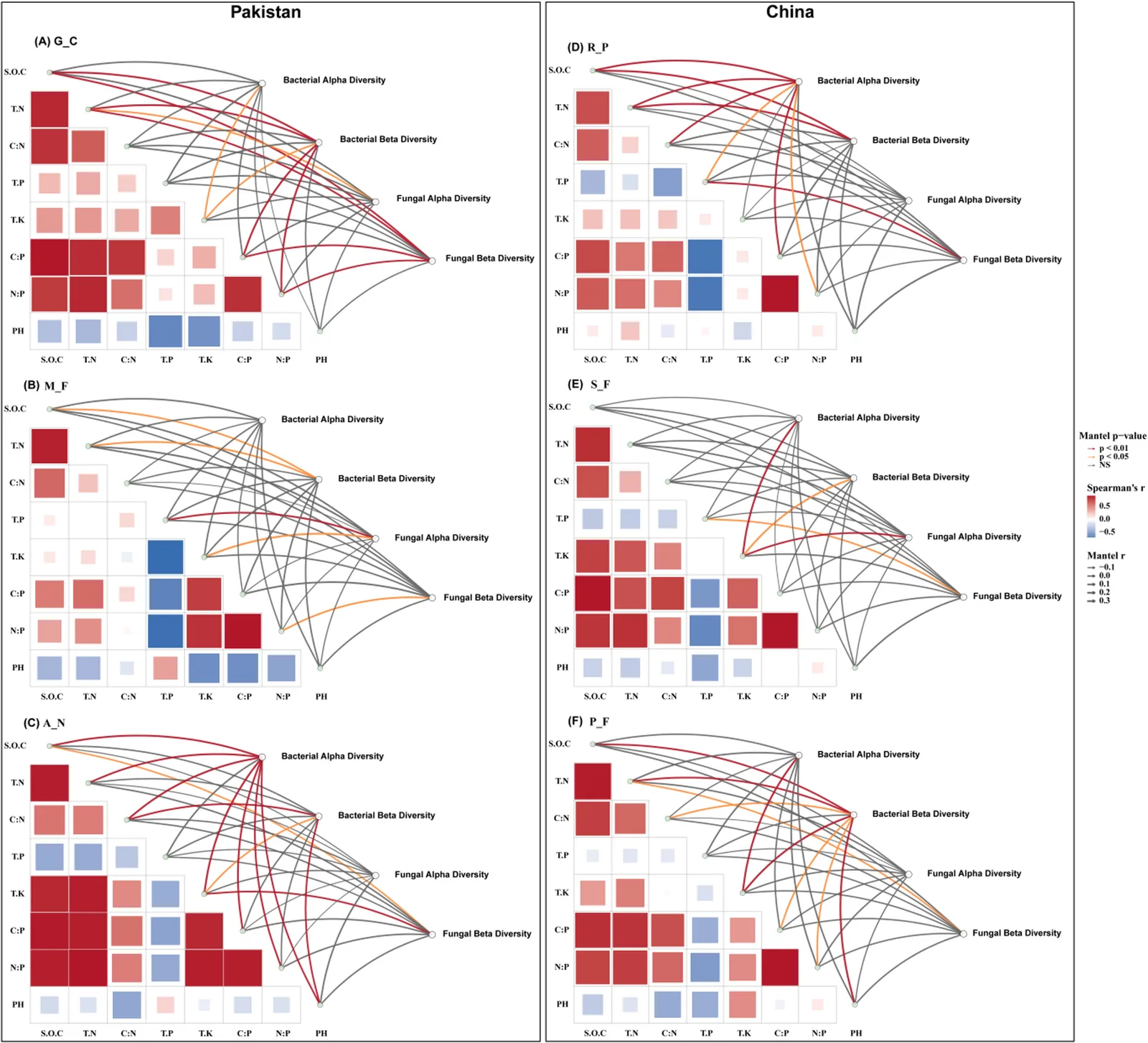
Mantel analysis of soil properties’ influence on microbial diversity and community composition across successional stages in Pakistan and China (*n* = 18)

**Fig. 5:**
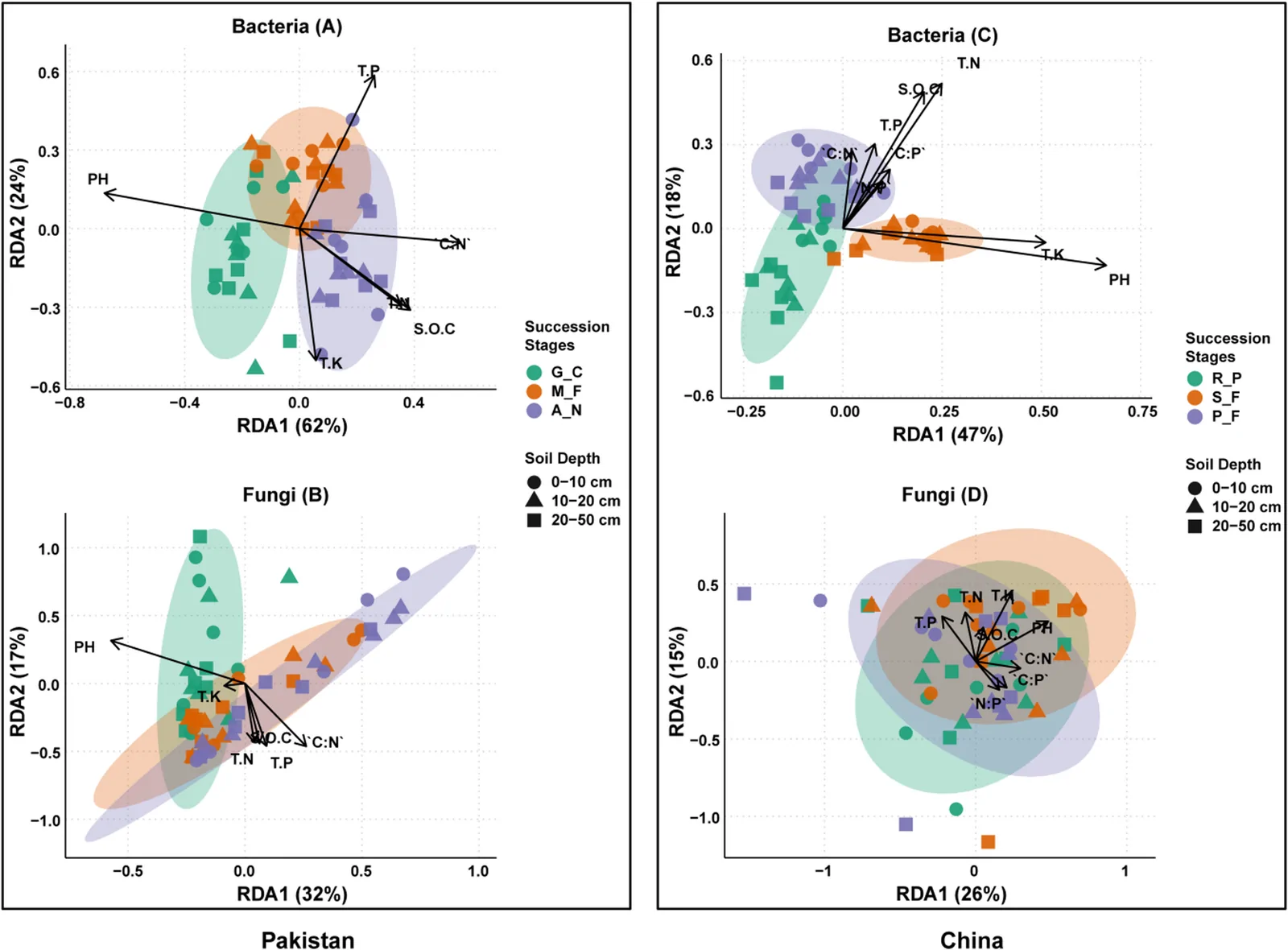
Redundancy analysis (RDA) of bacterial and fungal communities in soil samples from Pakistan and China (*n* = 18) forest succession stages. (**A**) Bacterial community composition in Pakistan. (**B**) Fungal community composition in Pakistan. (**C**) Bacterial community composition in China. (**D**) Fungal community composition in China. Key: Data points represent individual soil samples, with shapes indicating soil depth (■ 0–10 cm, ▲ 10–20 cm, ▼ 20–50 cm) and colors indicating successional stages

The relationship between soil physicochemical properties and microbial diversity and composition was assessed using Mantel tests and redundancy analysis (RDA) across forest succession stages in Pakistan and China (Figs. 4 and 5). Across all sites, forest succession was associated with changes in the relationships between microbial communities and soil physicochemical properties across stages in both countries. In early succession, bacterial and fungal diversity showed significant correlations to SOC, TN, TP, and nutrient ratios (C: P, N: P; Mantel *r* = 0.23–0.43, *p* < 0.01), In mid-successional stage, bacterial diversity was correlated with SOC, TN, and TK, and fungal diversity was correlated with TP, TK, and nutrient ratios. In late succession, bacterial diversity was significantly associated with SOC, TN, C:N, TK, C:P, N:P, and pH (*r* = 0.34–0.66, *p* < 0.01). Fungal responses were weaker at this stage, with only moderate correlations with SOC, TN, and TK.

RDA results were consistent with Mantel test outcomes. Bacterial community composition showed weaker associations with soil properties in early and mid-successional stages, but clearer alignment with TK, pH, C:N, and C: P in late-successional stages in both countries. Fungal community composition showed limited alignment with soil variables in early succession and stronger associations with SOC, TN, TP, C:P, and pH in late-successional stages.

Panels A, B, and C show network diagrams for the Pakistani successional stages (G_C, M_F, A_N), illustrating the relationships between soil properties and microbial diversity (alpha diversity and beta diversity). Panels D, E, and F show the Chinese successional stages (R_P, S_F, P_F). The thickness of the lines represents the strength of the correlation, with wider lines indicating stronger correlations from the Mantel test’s r value. The color of the lines reflects statistical significance based on 999 permutations: red for positive correlations, orange for negative correlations, and gray for non-significant correlations (NS).

## Discussion

### Soil Physicochemical Dynamics during Forest Succession in Pakistan and China

Our results reveal a distinct biogeochemical pattern, influenced by variations in soil development, climate and dynamics of the vegetation, which act as environmental filters shaping microbial succession in both regions. In Pakistan, SOC and TN increased in late succession, consistent with global patterns of organic matter accumulation in mature forests [24]. The pronounced decline in soil pH indicates progressive soil acidification, commonly associated with organic matter accumulation and the depletion of base cations. In late succession stage (A_N) in Pakistan, both C: P and N: P ratios increased, indicating phosphorus limitation as the forests mature. These dynamics align with the Walker and Syers [25] model, which predicts that the availability of the phosphorus will decrease in the forest ecosystems with high organic matter accumulation rates.

In contrast, the stoichiometry of Chinese forests successional stages was more complex and region specific. High SOC concentrations were observed in the mid-successional (S_F), rather than in the late-successional (P_F), contrary to most common expectations [26]. This pattern may reflect transient changes in soil carbon dynamics during forest recovery. For example, differences in microbial community composition and litter quality between successional stages may influence carbon turnover rates, while temporary increases in plant-derived carbon inputs during mid-succession could also contribute to SOC accumulation. However, because root biomass, litter inputs, and decomposition rates were not directly measured in this study, these interpretations should be considered speculative and warrant further investigation. In Chinese successional stages, C:P and N: P ratios showed the opposite trend and were reduced in late successional stage (P_F). This suggests that the recycling of phosphorus in China, which could be regulated due to extensive mycorrhizal connections [27], or increased mineral weathering, is effective, and it helps sustain phosphorus in late-successional stages. The very low total potassium (TK) in PF (0.30 g/kg) suggests that K may be a co-limiting factor to the microbial activity and productivity in these highly weathered tropical forests [28].

TN levels increased with succession across both regions, which confirms that nutrient retention was higher in the late-successional stages as well [29]. During succession, the C: N ratio increased in both countries, suggests the accumulation of recalcitrant, lignin-rich organic materials that are more resistant to decomposition in mature forest ecosystems [30].

### Microbial Alpha Diversity across Succession and Stratification in Pakistani and Chinese Forest Succession Stages

Our results showed distinct biogeographic patterns in bacterial and fungal alpha diversity responses to forest succession in both Pakistan and China (Fig. 1; Table 1), highlighting strong associations with environmental conditions and successional history. In both regions, microbial alpha diversity showed its strongest variation across forest successional stages (Fig. 1), whereas vertical stratification effects were more pronounced in Chinese forest successional stages. In addition, the trend in the bacterial and fungal diversity diverged with the succession, suggesting contrasting ecological strategies at the kingdom level.

In Pakistan, the bacterial richness and diversity were highest at mid-successional stage (M_F) and were correlated with higher nutrient levels (e.g., total phosphorus), whereas fungal richness peaked in late-successional stage (A_N), where C: N ratios were highest. A similar succession pattern was observed in China, where bacterial diversity peaked at mid-successional (S_F), and fungal diversity was the highest in the late-successional (P_F), particularly the deeper soil [31]. This pattern is consistent with resource pulse hypothesis, which states that bacterial diversity positively correlates with moderate levels of disturbance and nutrient heterogeneity [32]. The later successional stages were predominantly dominated by fungal communities, particularly symbiotic and saprotrophic taxa commonly associated with recalcitrant organic substrates and increased vegetation complexity [33]. The different peaks in bacterial and fungal diversity suggest that their successional dynamics are not synchronized. The highest bacterial diversity observed in mid-succession in both regions suggests an association with fluctuating nutrient availability and increased habitat heterogeneity [34]. In contrast, fungal diversity increased toward later successional stages, a pattern commonly associated with greater accumulation of complex carbon substrates and more stable plant–fungal associations [35]. This observation suggests that fungal community diversity may be more closely associated with carbon quality (e.g., high C: N ratios) than with carbon quantity alone [36].

Importantly, the strength and pattern of associations between soil properties and microbial diversity varied across successional stages, consistent with our second hypothesis. In early-successional stages of both regions, both bacterial and fungal beta diversity showed strong positive correlations with many of the major soil properties, including soil organic carbon (SOC), total nitrogen (TN), and nutrient ratios such as C: P and N: P (Mantel test, Fig. 4). These findings are different from those found in more disturbed environments, where weaker correlations may be more common [37]. Bacterial alpha diversity was specifically related to total potassium (TK) (Mantel *r* = 0.18). The strong, generalized correlations with SOC, TN, C:P, and N: P in this stage suggest that nutrient availability is closely associated with microbial community structure during early forest succession. Conversely, the late-successional stage of both regions also exhibited significant, multifactorial relationships between fungal and bacterial diversity and major soil variables, such as soil organic carbon (SOC), total nitrogen (TN), C:N ratio, total potassium (TK), and pH (Mantel *r* = 0.34–0.58) [38]. This pattern is consistent with the notion that maturing forest systems exhibit stronger and more predictable associations between soil properties, nutrient cycling, and microbial communities [39]. A distinct trend was noted in the mid-successional stage in both regions. In this stage, TK showed significant relationships with bacterial diversity, whereas fungal diversity showed no significant correlations with soil properties. This apparent mid-successional decoupling may be associated with transient functional redundancy or niche restructuring, particularly within tropical forests that are the result of a historically recent disturbance such as the use of rubber plantations [40].

Pakistani soils showed no significant vertical stratification in bacterial or fungal alpha diversity across the 0–50 cm soil profile (Fig. 1; Table 1). This uniform pattern may be associated with broad environmental conditions such as arid climate and neutral to alkaline soil pH [41, 42], as well as relatively uniform distributions of total potassium and phosphorus along the soil profile (Table S1). However, because we did not directly measure soil moisture, root biomass, or microbial activity by depth, these interpretations remain speculative. The observed pattern could also result from composite sampling across the 0–50 cm profile and the limited number of replicate samples per depth (*n* = 6), which together reduce the resolution and statistical power to detect finer-scale vertical differences in microbial communities. This contrasted with Chinese forest succession stages, which exhibited distinct vertical stratification, as bacterial richness declined with depth (e.g., RP Chao1: 15.06 at 0–10 cm to 9.04 at 20–50 cm), whilst fungal diversity in PF similarly increased with depth (Fig. 1), benefiting from increased organic matter and humification in deeper soils. Such trends may be related to more pronounced vertical shifts in soil organic carbon (SOC) and pH (pH < 5.0 at all stages), characteristic of humid tropical soils that tend to undergo active leaching and accumulate organic acids [43, 44]. Overall, successional stage was relevant across regions, whereas regional climate and soil chemistry were associated with differences in the expression of vertical patterns of microbial diversity [45].

### Microbial Community Composition and Soil Interactions during Forest Succession

Our observed patterns support our hypotheses that microbial community composition varies across forest successional stages (Figs. 2 and 3, Table S2), and that the strength of associations between microbial communities and soil properties differs among successional stages (Fig. 5 & Fig. S2). The observed patterns indicate that environmental filters, particularly soil geochemistry (pH, SOC, C:N), nutrient availability, and successional history are closely associated with variation in microbial taxonomic composition, with potential implications for belowground ecosystem functioning and resilience [46]. Such change is consistent with the resource-acquisition trade-off hypothesis, according to which the copiotrophic taxa, which inhabit nutrient-poor soils with high pH levels, are slowly replaced by the oligotrophic taxa that are more adapted to acidic, organic-rich soils [34].

In Pakistani forest succession stages, bacterial communities shifted from copiotrophic groups toward oligotrophic groups across the forest successional stages. Actinobacteriota was most abundant in the early-successional stage (G_C), which coincided with relatively high soil pH and lower C: N and C: P ratios, conditions commonly associated with rapid colonizers and r-strategists (copiotrophs) that exploit labile carbon. With increasing SOC, TN, and nutrient stoichiometry (C: N, C: P, N: P) across succession, the relative abundance of *Acidobacteriota* and *Proteobacteria* increased, consistent with their oligotrophic lifestyles and adaptation to nutrient-poor, recalcitrant substrates [47, 48]. The progressive decline in soil pH across succession in Pakistan (from 8.21 in G_C to 5.66 in A_N), may have contributed to this compositional shift as *Acidobacteriota* are favored in acidic environments, leading to more stable microbial assemblages in late-successional soils [49, 50]. This stabilization may be associated with the accumulation of recalcitrant organic matter, which restricts access to labile nutrients, and an increasing complexity of microbial interactions.

Bacterial community composition in Pakistan showed strong associations with soil pH (Fig. 5), and this is consistent with global findings that soil pH has been widely reported as an important correlate of bacterial niche partitioning, potentially through its effects on mineral solubility and ion toxicity [50]. Similar trends are observed in other ecosystems such as fynbos soils, where *Acidobacteriota* shows high relative abundance [51]. These findings are consistent with the classical model presented by Odum [52], as ecosystems mature, they often exhibit more structured and predictable associations between soil properties and biological communities.

In contrast, Chinese forest succession stages showed a more complex successional pattern. Here, SOC, TK, and soil pH first increased from early (R_P) to mid-successional stage (S_F) and then declined in late succession (P_F). This “hump-shaped” pattern in soil resources was reflected by the bacterial community, where *Actinobacteriot*a declined continuously and *Acidobacteriota* and *Chloroflexi* first decreased and then increased across succession. This pattern suggests that mid-successional stages in China were associated with conditions (moderate SOC and nutrient availability) that favored copiotrophic growth [47]. The later decline in SOC and TK, however, coincided with an increase in oligotrophic *Acidobacteriota* and *Chloroflexi*, particularly in deeper soils, suggesting that soil depth and nutrient stratification were more strongly associated with bacterial composition in Chinese than in Pakistani forest succession stages [53].

In Chinese forest succession stages the total potassium (TK) and soil pH emerged as strong edaphic correlates of bacterial community composition (Fig. 5), particularly in more acidic late successional stage (pH 4.17). This observation is consistent with the studies indicating that soil characteristics, including potassium and pH seem to significantly impact the microbial community composition during forest succession [54]. Under acidic conditions, potassium leaching may be associated with shifts in microbial community dynamics, particularly in soils with low pH is low, because the dominant bacterial phyla (e.g., *Acidobacteriota*) rely on potassium to regulate their osmotic equilibrium and perform metabolic processes [51]. *Acidobacteriota*, which is abundant in low-potassium, acidic soils, may be relatively more abundant under potassium-limited and acidic conditions, potentially reflecting physiological traits related to osmotic regulation and stress tolerance in oligotrophic taxa.

Fungal community composition also exhibited successional trends that correspond with soil chemistry. In both countries, *Ascomycota* dominated early to mid-successional stages, aligning with their r-strategist lifestyle and ability to exploit simple carbon compounds and cellulose from accumulating litter [55]. Their peak abundance in mid-succession coincided with increasing SOC and TN, indicating that abundant litter input provided substrates favorable for *Ascomycota*. By contrast, *Basidiomycota*, many of which are lignin decomposers or ectomycorrhizal fungi, became more abundant in late succession and with increasing soil depth. This pattern is consistent with the accumulation of recalcitrant organic matter (high C: N and C: P ratios) and decreasing pH, conditions that favor K-strategists with specialized enzymatic machinery for decomposing complex polymers [56]. Notably, in Chinese forest succession stages, soil depth effects were stronger, with *Basidiomycota* dominating at deeper layers (20–50 cm), whereas in Pakistani soils, *Ascomycota* remained dominant across all depths. Geographical variation highlights the importance of soil depth in determining fungal community structure, with deeper soils being more favorable to *Basidiomycota*. These findings suggest that fungal responses to succession are not uniform but are mediated by interactions between soil nutrient stoichiometry, substrate quality, and vertical nutrient gradients.

The fungal communities had higher regional and depth stage-specific responses. Fungal composition in Pakistani forests was significantly related to SOC and C: N (Fig. 5 & Fig. S2). The fungi are known to be ligninolytic specialists and have high carbon requirements and are closely associated with soil carbon dynamics during forest succession [57]. Conversely, Chinese forest succession stages did not show a clear relationship between fungal communities and the measured soil properties (Fig. 5 & Fig. S2), wherein the fungal community may be more strongly influenced by abiotic factors (e.g., host tree associations) or biotic properties (e.g., mycorrhizal networks), especially on the tropical soils that are phosphorus-limited and exhibit acidic soil properties [58]. This variation highlights the importance of biotic processes (i.e., symbiosis), which can be more prominent in shaping fungal communities in the tropical forests than in the semi-arid ones, as in Pakistan.

While our results mostly follow the classic pattern of copiotrophs being replaced by oligotrophs, more recent studies reveal that microbial strategies are dynamically sensitive to changes in nutrient availability, pH and history of disturbance. For example, *Actinobacteriota* can grow like copiotrophs when nutrients are high or soils are disturbed [47, 59], and some *Acidobacteria* and *Proteobacteria* act like oligotrophs in nutrient-poor soils. This microbial flexibility suggests that the functional role of microbial taxa can change with changes in resource availability or disturbance history, enabling these microbial groups to persist under a wide range of environmental conditions. Since the forest succession stages in our experiment underwent consistent succession without severe disturbances, the gradual change in the community structure may be the reason why we found the common reduction in *Actinobacteriota* and growth in *Proteobacteria*. These results indicate that microbial trophic strategies are highly adaptive and responsive to fluctuations in soil nutrients, pH, and disturbance history, which ultimately affect ecosystem processes including nutrient cycling and organic matter decomposition.

Based on these results, the semi-arid Pakistan region soil chemistry becomes increasingly important as the forest age advances, and the pH becomes the key ecological filter [60, 61]. The RDA plots showed that pH was the environmental variable most strongly associated with the separation of alkaline-adapted bacterial communities in G_C and acid-tolerant assemblages in A_N, highlighting the strong association between pH and microbial community composition due to nutrient solubility and the community’s metabolic boundaries [62]. Conversely, TK explained a large proportion of the constrained variation in bacterial composition in Chinese forest succession stages, and its association with nutrient cycling highlights the potential importance of potassium availability in microbial functioning within nutrient-limited and leached tropical systems [63]. Such trends are further regulated by the climatic limitations: vertical pH gradients and reduction in organic matter turnover are intensified by aridity in Pakistan, whereas nutrient leaching and stratification are intensified by humidity in China [41, 45].

Overall, our results confirmed that forest succession emerged as the dominant gradient associated with microbial community composition in both Pakistan and China, with soil properties acting as the main environmental filters. In Pakistan, the progressive decline in soil pH was strongly associated with variation in bacterial assemblage composition, reinforcing the shift from copiotrophic to oligotrophic taxa and potentially slowing decomposition rates in late-successional stages by restricting nutrient release from litter [64]. In China, potassium limitation and soil nutrient stratification emerged as key factors associated with bacterial composition, especially in late succession, which may be linked to altered microbial nutrient recycling, with potential consequences for forest productivity and carbon stabilization [65]. To integrate the observed patterns of microbial alpha diversity and community composition, as well as soil depth effects and soil–microbe associations identified by NMDS, RDA, and Mantel analyses, we provide a conceptual graphical synthesis in Supplementary Figure S1. These patterns support our hypotheses that microbial composition shifts across succession and that the strength of microbe–soil relationships varies with successional stage, being weaker during transitional mid-successional stages.

## Conclusion

Our study shows that forest succession in Pakistan and China strongly shapes soils and microbial communities composition. As forests mature, soils generally accumulate more carbon and nitrogen, become more acidic, and shift in nutrient balance, with some regional differences. In Pakistan, succession follows a steady pattern, with soils enriching in organic matter and microbial communities transitioning toward taxa adapted to increasingly stable and resource-regulated soil conditions. Community composition shifts along succession in response to changes in soil resources, pH, and ecosystem stability, with dominant microbial taxa exhibiting adaptive and context-dependent strategies rather than fixed copiotrophic or oligotrophic roles. In Chinese forest succession stages, similar trends occur, but soil properties and microbial dynamics are influenced by tropical climate and past disturbances. Microbial diversity shows different trends, bacteria peak in mid-succession under nutrient-rich and heterogeneous soils, while fungi thrive in late stages supported by recalcitrant organic matter and plant associations. Community composition shifts accordingly, from copiotrophs in early stages to oligotrophs and specialized decomposers in late stages. These findings demonstrate that forest succession drives not only aboveground vegetation changes but also belowground transformations, enriching soils and stabilizing microbial communities. Protecting late-successional stage is critical, as they serve as reservoirs of soil carbon and microbial diversity, supporting nutrient cycling, ecosystem stability, and climate resilience. Further studies should also focus on understanding the assembly and co-occurrence patterns of microbial communities across different forest successional stages, as these interactions will help uncover the underlying mechanisms driving microbial diversity and ecosystem processes.

## Supplementary Information

Below is the link to the electronic supplementary material.

Supplementary Material 1 (DOCX 1.18 MB)

## Data Availability

The datasets generated during and/or analysed during the current study are available from the corresponding author on reasonable request.
