# Supplementary material for: Forest Succession Shapes Soil microbial Communities through Region-specific Edaphic Filters in Tropical and Subtropical Forests
Source: Microb Ecol. 2026 Mar 23;89(1):90. doi: 10.1007/s00248-026-02734-1 (PMC13053364; doi:10.1007/s00248-026-02734-1)
Supplement: Supplementary file 1 — Supplementary Material 1 (DOCX 1.18 MB) [file 248_2026_2734_MOESM1_ESM.docx]

**Supplementary Data**

**Forest succession shapes soil microbial communities through region-specific edaphic filters in subtropical and tropical forests**

Waseem Muhammad^1,2,3^, Xinyu Zhou^1,2,3,4^, Xiaocheng Yu^1,2,3^, Kechen Yang^1,2,3^, Rong Bai^1,2,3^, Leiyun Feng^1,2,3,5^, Qiang Luo^1,2,3^, Zhiqing Zhou^1,2,3^ , Cong Wang^6^, Jingchao Li^1,2,3^, Kui Ji^7^, Hua-Zheng Lu^1,2,3,4,5*^

^1^ Yunnan Key Laboratory of Forest Ecosystem Stability and Global Change, Xishuangbanna Tropical Botanical Garden, Chinese Academy of Sciences, Mengla, China

^2^ Xishuangbanna Station for Tropical Forest Ecosystem Studies, Chinese Academy of Sciences, Mengla, China

^3^School of Life Science, University of Chinese Academy of Sciences, Beijing China

^4^ College of Landscape Architecture and Forestry, Shandong Key Laboratory for Germplasm Innovation of Saline‐Alkaline Tolerant Grasses and Trees, Qingdao Agricultural University, Qingdao, P.R. China

^5^ Schools of Soil and Water Conservation, Southwest Forestry University, Kunming, China

^6^ Institutes of Microbiology, Chinese Academy of Sciences, Beijing, China

^7^ Xishuangbanna National Nature Reserve, Mengla 666300, China

*Correspondence author email: [luhuazheng@xtbg.ac.cn](mailto:luhuazheng@xtbg.ac.cn)

**Table S1.** Soil physicochemical properties (mean ± SD) across forest succession stages and soil depths (0–10 cm, 10–20 cm, 20–50 cm) in Pakistan (G_C: artificial forest, M_F: secondary forest, A_N: primary forest) and China (R_P: rubber plantation, S_F: secondary rainforest, P_F: primary forest) (n = 6). Variables include soil organic carbon (SOC), total nitrogen (TN), carbon-to-nitrogen ratio (C:N), total phosphorus (TP), total potassium (TK), carbon-to-phosphorus ratio (C:P), nitrogen-to-phosphorus ratio (N:P) and pH. Different lowercase letters within a forest succession and soil property indicate significant differences (p < 0.05) among soil depths, based on Tukey’s HSD post-hoc test. Asterisks in the rightmost column indicate significance of forest type effect from ANOVA tests: **p < 0.001.

| **Region** | **Stages** | **Depth** | **SOC (Mean ± SD, g/kg)** | **TN (Mean ± SD, g/kg)** | **C:N (Mean ± SD, g/kg)** | **TP (Mean ± SD, g/kg)** | **TK ((Mean ± SD, g/kg)** | **C:P (Mean ± SD, g/kg)** | **N:P (Mean ± SD, g/kg)** | **pH** | **Stages Significance** |
| --- | --- | --- | --- | --- | --- | --- | --- | --- | --- | --- | --- |
| **Pakistan** | G_C | 1 –10 cm  10- 20 cm  20 –50 cm | 4.77 ± 1.76 (a)  4.31 ± 2.57 (a)  4.25 ± 2.60 (a) | 0.73 ± 0.18 (a)  0.68 ± 0.24 (a)  0.72 ± 0.24 (a) | 6.41 ± 1.2 (a)  5.94 ± 1.68 (a)  5.57 ± 1.8 (a) | 14.39 ± 1.90(a)  14.47 ± 1.01(a)  14.20 ± 1.32(a) | 0.59 ± 0.09 (a)  0.62 ± 0.08 (a)  0.60 ± 0.08 (a) | 0.33 ± 0.12 (a)  0.29 ± 0.17 (a)  0.3 ± 0.18 (a) | 0.05 ± 0.01 (a)  0.05 ± 0.02 (a)  0.05 ± 0.02 (a) | 8.21 ± 0.30 (a)  7.97 ± 0.25 (a)  7.98 ± 0.16 (a) | *** |
|  | M­_F | 1 –10 cm  10- 20 cm  20 –50 cm | 6.91 ± 2.59 (a)  6.89 ± 1.47 (a)  7.00 ± 2.19 (a) | 0.84 ± 0.25 (a)  0.86 ± 0.19 (a)  0.91 ± 0.23 (a) | 8.01 ± 0.93 (a)  7.97 ± 0.47 (a)  7.69 ± 1.6 (a) | 27.62 ± 8.83(a)  26.72 ± 7.27(a)  27.56 ± 7.72(a) | 0.34 ± 0.25 (a)  0.35 ± 0.26 (a)  0.38 ± 0.29 (a) | 0.28 ± 0.13 (a)  0.27 ± 0.09 (a)  0.27 ± 0.09 (a) | 0.03 ± 0.01 (a)  0.04 ± 0.01 (a)  0.04 ± 0.01 (a) | 7.15 ± 0.15 (a)  7.13 ± 0.14 (a)  7.06 ± 0.14 (a) | *** |
|  | A_N | 1 –10 cm  10- 20 cm  20 –50 cm | 44.09 ± 8.44 (a)  36.63 ± 7.45 (a)  41.17 ± 6.96 (a) | 3.85 ± 3.31 (a)  3.39 ± 3.36 (a)  3.57 ± 2.85 (a) | 10.52 ± 2.22(a)  9.84 ± 2.45 (a)  10.82 ± 2.54(a) | 18.16 ± 2.94(a)  18.90 ± 1.61(a)  19.26 ± 1.90(a) | 0.73 ± 0.40 (a)  0.69 ± 0.40 (a)  0.70 ± 0.35 (a) | 2.52 ±2.31 (a)  2.08 ± 1.29 (a)  2.23 ± 1.2 (a) | 0.22 ± 0.2 (a)  0.19 ± 0.1 (a)  0.19 ± 0.1 (a) | 5.66 ± 0.21 (a)  5.76 ± 0.33 (a)  5.66 ± 0.31 (a) | *** |
| **China** | R_P | 1 –10 cm  10- 20 cm  20 –50 cm | 15.06 ± 2.59 (a)  12.17 ± 2.47 (b)  9.04 ± 1.96 (c) | 1.77 ± 0.29 (a)  1.46 ± 0.16(b)  1.25 ± 0.15 (c) | 8.78 ± 0.73(a)  8.41 ± 0.67(b)  7.4 ± 0.51 (c) | 6.70 ± 1.63 (a)  7.62 ± 1.95 (b)  8.61 ± 1.87 (c) | 0.31 ± 0.06 (a)  0.38 ± 0.18 (b)  0.30 ± 0.05 (c) | 2.33 ± 0.56 (a)  1.73 ± 0.69 (b)  1.13 ± 0.49 (c) | 0.28 ± 0.08 (a)  0.21 ± 0.06 (b)  0.15 ± 0.03 (c) | 4.12 ± 0.13 (a)  4.09 ± 0.13 (a)  4.06 ± 0.11 (a) | *** |
|  | S_F | 1 –10 cm  10- 20 cm  20 –50 cm | 23.15 ± 3.83 (a)  14.80 ± 3.03 (b)  9.91 ± 2.32 (c) | 2.42 ± 0.23 (a)  1.80 ± 0.23(b)  1.46 ± 0.23 (c) | 9.55 ± 1.06 (a)  8.25 ± 1.59(b)  6.94 ± 2.1 (c) | 9.71 ± 1.44 (a)  10.00 ± 1.7(b)  10.78 ± 2.49(c) | 0.55 ± 0.06 (a)  0.48 ± 0.04 (a)  0.41 ± 0.04 (b) | 2.45 ± 0.68 (a)  1.57 ± 0.68 (b)  0.99 ± 0.37 (c) | 0.25 ± 0.01 (a)  0.19 ± 0.01 (b)  0.14 ± 0.01 (c) | 5.63 ± 0.39 (a)  5.76 ± 0.25 (a)  5.74 ± 0.38 (a) | *** |
|  | P_F | 1 –10 cm  10- 20 cm  20 –50 cm | 22.41 ± 2.71 (a)  16.79 ± 2.37 (b)  11.70 ± 1.71 (c) | 2.55 ± 0.17 (a)  2.07 ± 0.17(b)  1.58 ± 0.18 (c) | 8.78 ± 0.73 (a)  8.1 ± 0.67 (ab)  7.4 ± 0.51 (a) | 11.35 ± 1.46(a)  12.21 ± 1.66(a)  12.89 ± 1.89(a) | 0.38 ± 0.06 (a)  0.35 ± 0.06 (a)  0.30 ± 0.08 (b) | 1.98 ± 0.16 (a)  1.39 ± 0.18 (b)  0.92 ± 0.17 (c) | 0.23 ± 0.02 (a)  0.17 ± 0.01 (b)  0.12 ± 0.02 (c) | 4.17 ± 0.24 (a)  4.19 ± 0.19 (a)  4.21 ± 0.19 (a) | *** |

**Table S2.** Summary of ADONIS, ANOSIM, and NMDS Results for Bacterial and Fungal Communities in Pakistan and China (n = 6).

| **Country** | **Biotic Communities** | **Variable** | **ADONIS** | | **ANOSIM** | | **Stress** |
| --- | --- | --- | --- | --- | --- | --- | --- |
|  |  |  | ***R²*** | **p-value** | ***R*** | **p-value** |  |
| **Pakistan** | **Bacteria** | Stages | 0.5262 | < 0.001 | 0.5677 | < 0.001 | 0.0998 |
|  |  | Depth | 0.4163 | > 0.05 | 0.03805 | < 0.05 |  |
|  | **Fungi** | Stages | 0.3491 | < 0.001 | 0.5192 | < 0.001 | 0.1296 |
|  |  | Depth | 0.6508 | > 0.05 | -0.0567 | > 0.05 |  |
| **China** | **Bacteria** | Stages | 0.7912 | < 0.001 | 0.5320 | < 0.001 | 0.07803 |
|  |  | Depth | 0.2089 | > 0.05 | 0.1812 | < 0.001 |  |
|  | **Fungi** | Stages | 0.5213 | < 0.001 | 0.4534 | < 0.001 | 0.1380 |
|  |  | Depth | 0.4786 | > 0.05 | 0.0774 | < 0.001 |  |

**Interpretation of R² and R values:**

- R² shows how much of the data variation can be explained by the factor (Stages, Depth).
- R shows how much the factor (Stages, Depth) differ from each other.

**Text S1.** The taxonomic profiles, Venn diagram analysis (Fig. S1) revealed substantial differences in the number of shared and unique operational taxonomic units (OTUs) among successional stages within each country. In Pakistan, the bacterial communities (Fig. S1A) showed a large core microbiome of 496 shared OTUs across all successional stages, indicating considerable overlap. However, each successional stage also hosted unique bacterial OTUs, with the early succession stage (G_C) contributing 151 unique OTUs, mid succession stage (M_F) contributing 80 unique OTUs, and late succession stage (A_N) contributing 169 unique OTUs. Similarly, fungal communities in Pakistan (Fig. S1B) exhibited 127 shared OTUs across all successional stages, with each successional stage maintaining distinct fungal OTUs: 324 unique to G_C, 49 unique to M_F, and 123 unique to A_N. In contrast, Chinese forest successional stages demonstrated both a large shared core and higher unique diversity in microbial communities. The bacterial communities (Fig. S1C) had 455 OTUs shared across all successional stages, with early (R_P), mid (S_F), and late (P_F) succession stages contributing 47, 275, and 64 unique OTUs, respectively. For fungal communities (Fig. S1D), 237 OTUs were shared across all successional stages, but unique OTU richness was notably higher compared to Pakistan: 97 OTUs were unique to R_P, 126 to S_F, and 134 to P_F. These patterns indicate that microbial community composition in Chinese forest successional stages is not only diverse but also contains a substantial number of habitat-specific taxa, particularly in fungal communities.


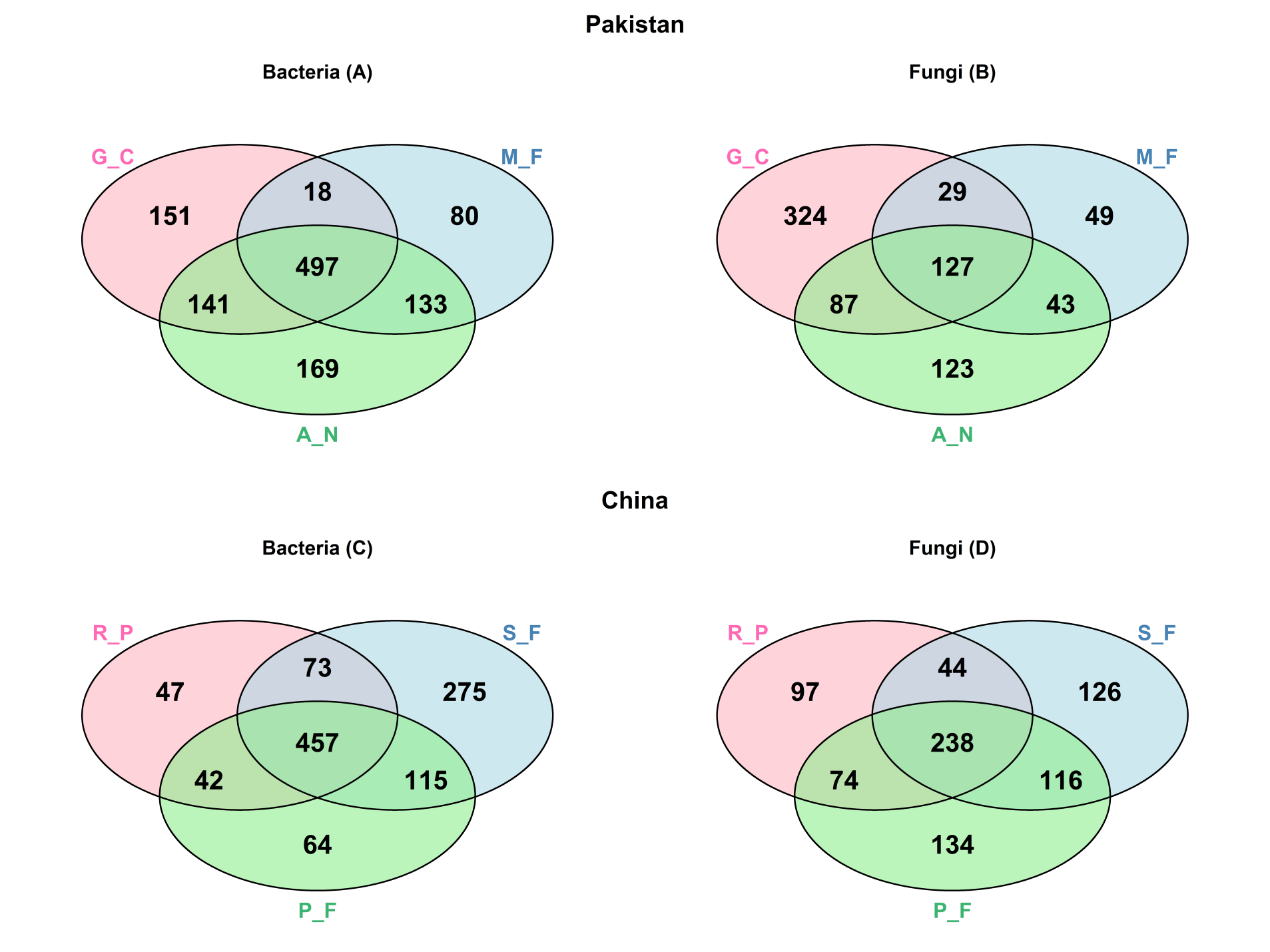


Figure S1. Microbial community overlap across successional stages in Pakistan and China (n = 18). (A) Bacterial OTU distribution among early (G_C), mid (M_F), and late (A_N) successional stages in Pakistan. (B) Fungal OTU distribution among early (G_C), mid (M_F), and late (A_N) successional stages in Pakistan. (C) Bacterial OTU distribution among early (R_P), mid (S_F), and late (P_F) successional stages in China (D) Fungal OTU distribution among early (R_P), mid (S_R), and late (P_F) successional stages in China.


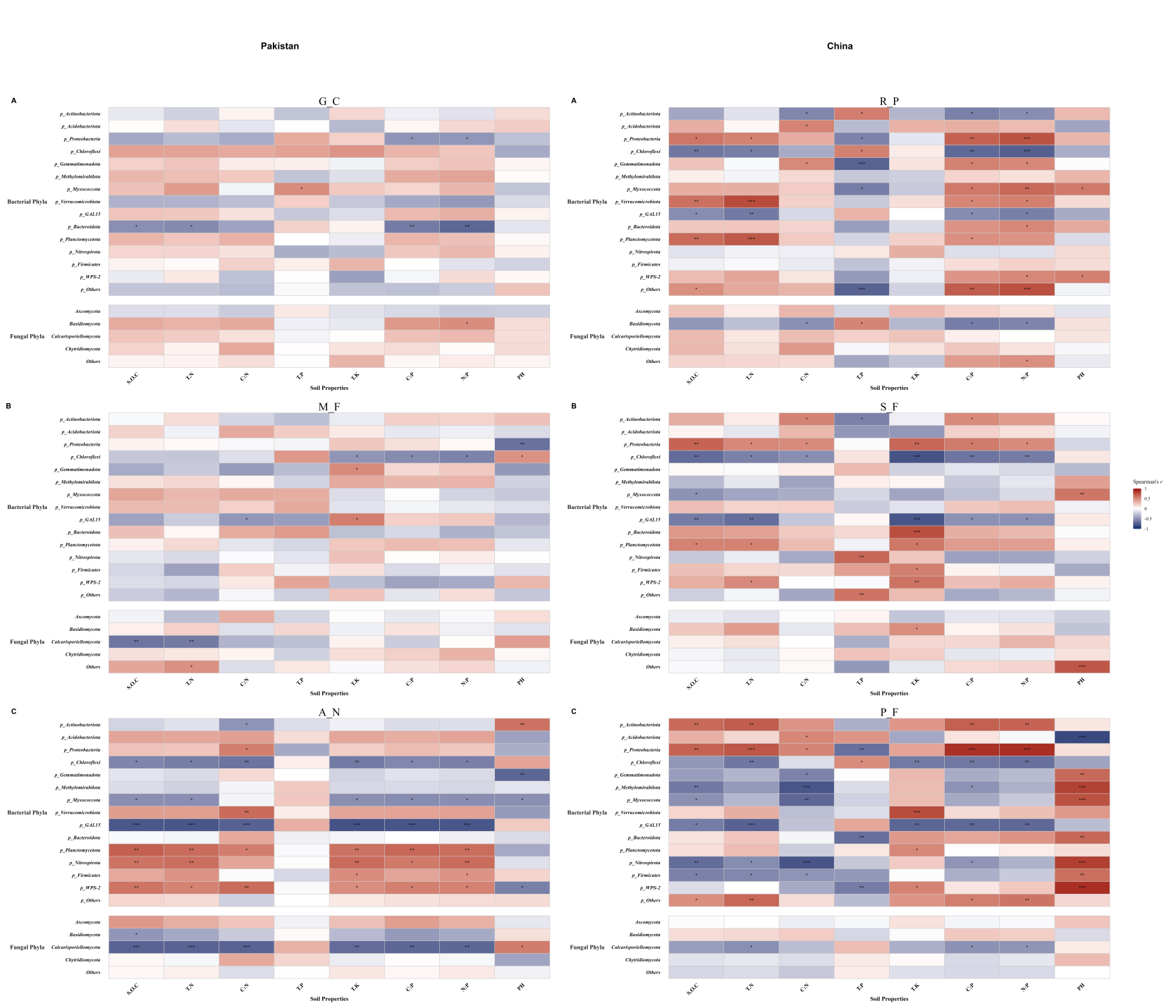


Figure S2. Heatmap analysis of soil properties' influence on microbial community characteristics across successional stages in Pakistan and China (n = 18). Panels A, B, C: Heatmaps showing Spearman correlation coefficients between soil properties and the composition of bacterial and fungal communities for G_C, M_F, and A_N Pakistani forest successional stages. Panels A, B, C: Heatmaps showing spearman correlation coefficients between soil properties and the composition of bacterial and fungal communities for R_P, S_F, and P_F Chinese forest successional stages, respectively. Each cell's color intensity reflects the strength of the correlation, with red indicating positive correlations and blue indicating negative correlations. The significance of correlations is marked with asterisks: * for p < 0.05 and ** for p < 0.01.

**
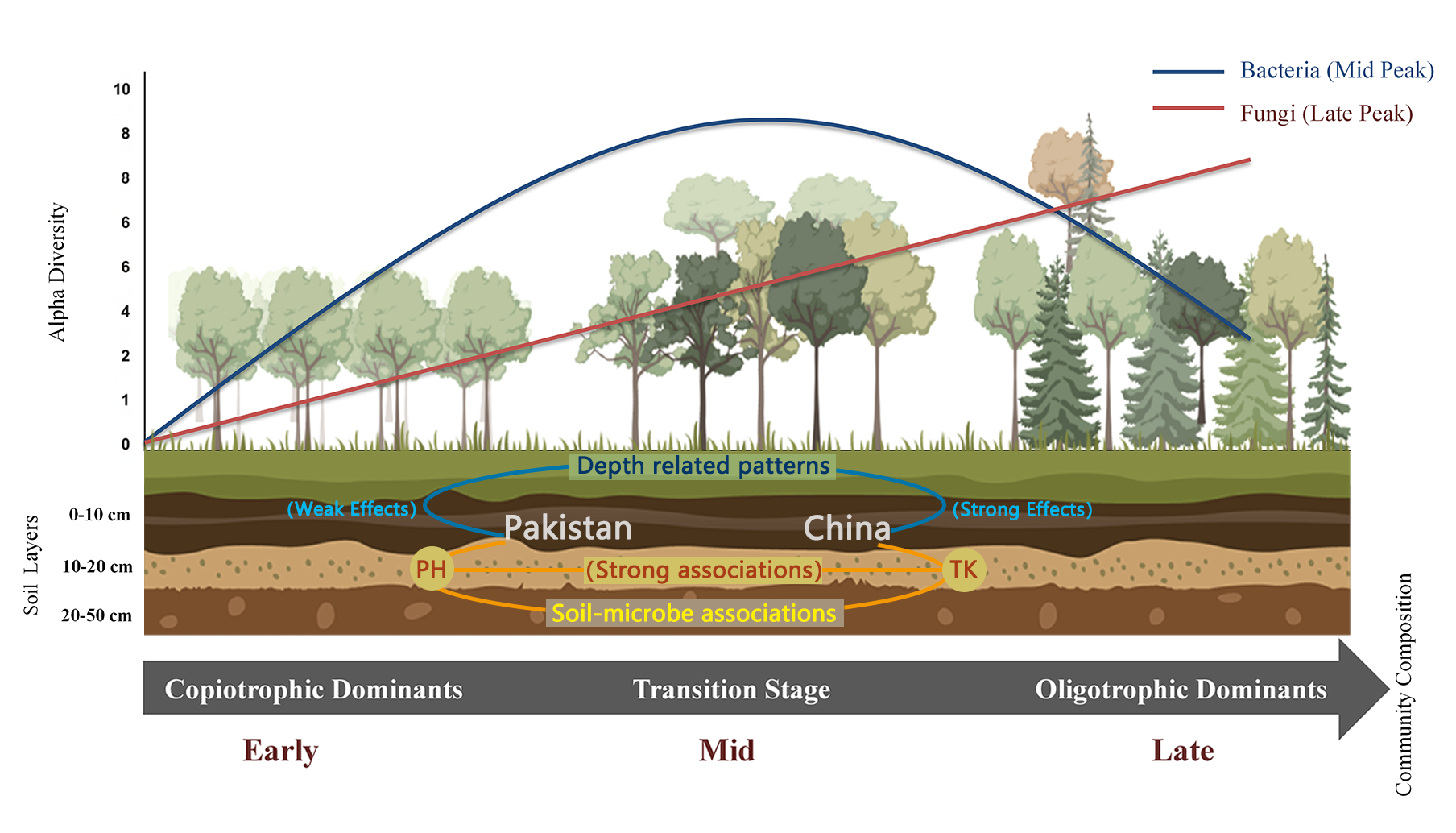
Figure S3: Conceptual graphical synthesis of microbial diversity patterns, community composition shifts, and soil–microbe associations across forest succession and soil depths.**

This figure integrates the main results of the study by summarizing: (i) observed alpha-diversity trends of bacterial and fungal communities across successional stages; (ii) shifts in microbial community composition from copiotrophic to oligotrophic dominance; (iii) region-specific, depth-related variation in microbial diversity and soil properties; and (iv) associations between key soil properties (e.g., pH in Pakistan and total potassium in China) and microbial communities. The schematic is based on outcomes from alpha-diversity analyses, NMDS ordination, redundancy analysis (RDA), and Mantel tests, and is intended to provide a visual synthesis of observed associative patterns.
